# High frequency of mosaic pathogenic variants in genes causing epilepsy-related neurodevelopmental disorders

**DOI:** 10.1038/gim.2017.114

**Published:** 2017-08-24

**Authors:** Mary Beth Stosser, Amanda S Lindy, Elizabeth Butler, Kyle Retterer, Caitlin M Piccirillo-Stosser, Gabriele Richard, Dianalee A McKnight

**Affiliations:** 1 GeneDx, Gaithersburg, Maryland, USA; 2 University of Chicago, Chicago, Illinois, USA

**Keywords:** epilepsy, genetic testing, mosaicism, next-generation sequencing, neurodevelopmental disorders

## Abstract

**Purpose:**

Mosaicism probably represents an underreported cause of genetic disorders due to detection challenges during routine molecular diagnostics. The purpose of this study was to evaluate the frequency of mosaicism detected by next-generation sequencing in genes associated with epilepsy-related neurodevelopmental disorders.

**Methods:**

We conducted a retrospective analysis of 893 probands with epilepsy who had a multigene epilepsy panel or whole-exome sequencing performed in a clinical diagnostic laboratory and were positive for a pathogenic or likely pathogenic variant in one of nine genes (*CDKL5*, *GABRA1*, *GABRG2*, *GRIN2B*, *KCNQ2*, *MECP2*, *PCDH19*, *SCN1A*, or *SCN2A*). Parental results were available for 395 of these probands.

**Results:**

Mosaicism was most common in the *CDKL5*, *PCDH19*, *SCN2A*, and *SCN1A* genes. Mosaicism was observed in *GABRA1*, *GABRG2*, and *GRIN2B*, which previously have not been reported to have mosaicism, and also in *KCNQ2* and *MECP2*. Parental mosaicism was observed for pathogenic variants in multiple genes including *KCNQ2*, *MECP2*, *SCN1A*, and *SCN2A.*

**Conclusion:**

Mosaic pathogenic variants were identified frequently in nine genes associated with various neurological conditions. Given the potential clinical ramifications, our findings suggest that next-generation sequencing diagnostic methods may be utilized when testing these genes in a diagnostic laboratory.

## Introduction

The development and adoption of next-generation sequencing (NGS) based diagnostics have revolutionized the ability to simultaneously analyze multiple genes in an accurate and efficient manner. Compared with traditional sequencing methods, the digital nature of NGS technology allows quantification of sequence variants such that a high read depth can enable the detection of low-level mosaic variants. As diagnostic NGS is increasingly used for sequence-based variant detection, there is mounting evidence that somatic and gonosomal mosaicism are more common and play a greater role in human genetic disorders than previously recognized. In affected patients, enhanced detection of somatic mosaicism can provide a definitive molecular diagnosis to patients who may have received negative results from less sensitive testing and can also directly affect disease management.^1^ Additionally, the detection and reporting of mosaicism is relevant to the families of probands for accurate estimates of recurrence risk. When mosaicism is detected in apparently healthy parents of an affected child, it is essential for assigning a more accurate recurrence risk, where that risk would increase from negligible for a true de novo variant to up to the published theoretical risk of 33%.^2^

Mosaicism can be classified into three types depending on the affected stage of development and the affected tissue(s): germ-line mosaicism, somatic mosaicism, and gonosomal mosaicism (a combination of germ-line and somatic mosaicism).^3^ Owing to limitations in tissue sampling, it is difficult to precisely identify the type of mosaicism in all situations. Several genetic disorders often involve mosaicism, for example, neurofibromatosis type 1, Proteus syndrome, linear nevus sebaceous syndrome, McCune–Albright syndrome, Beckwith–Wiedemann syndrome, and tuberous sclerosis complex. Based on case reports, mosaicism also plays a role in other types of genetic disorders such as neurodevelopmental and epilepsy-related disorders.^4, 5, 6, 7, 8, 9, 10, 11^

To date, the frequency of mosaicism in neurodevelopmental disorders has been evaluated mostly in research studies of individuals with disorders such as autism and intellectual disability.^12, 13^ A systematic study of the role of mosaicism in epilepsy or other neurodevelopmental disorders, especially in a clinical diagnostic setting, is currently lacking. Although it has been hypothesized that the frequency of mosaicism in certain epilepsy-related genes may be higher than previously appreciated, no information is available regarding the overall frequency of mosaic pathogenic variants in specific genes or disorders associated with epilepsy.^4, 5, 6, 7, 8, 9, 10, 11^ Additionally, parental mosaicism has been reported in some epilepsy-related genes. For example, a high incidence (at least 7%) of parental mosaicism has been detected by direct sequencing in families with inherited *SCN1A*-related epilepsies. In these families, the parents included individuals who were asymptomatic, mildly affected, and severely affected, though this type of clinical information for other genes may not be available.^14^

In this study, we report for the first time the extent and level of mosaicism detectable by multigene or whole-exome NGS for patients with epilepsy undergoing testing in a clinical diagnostic laboratory.

## Materials and methods

### Patient population

**Figure 1:**
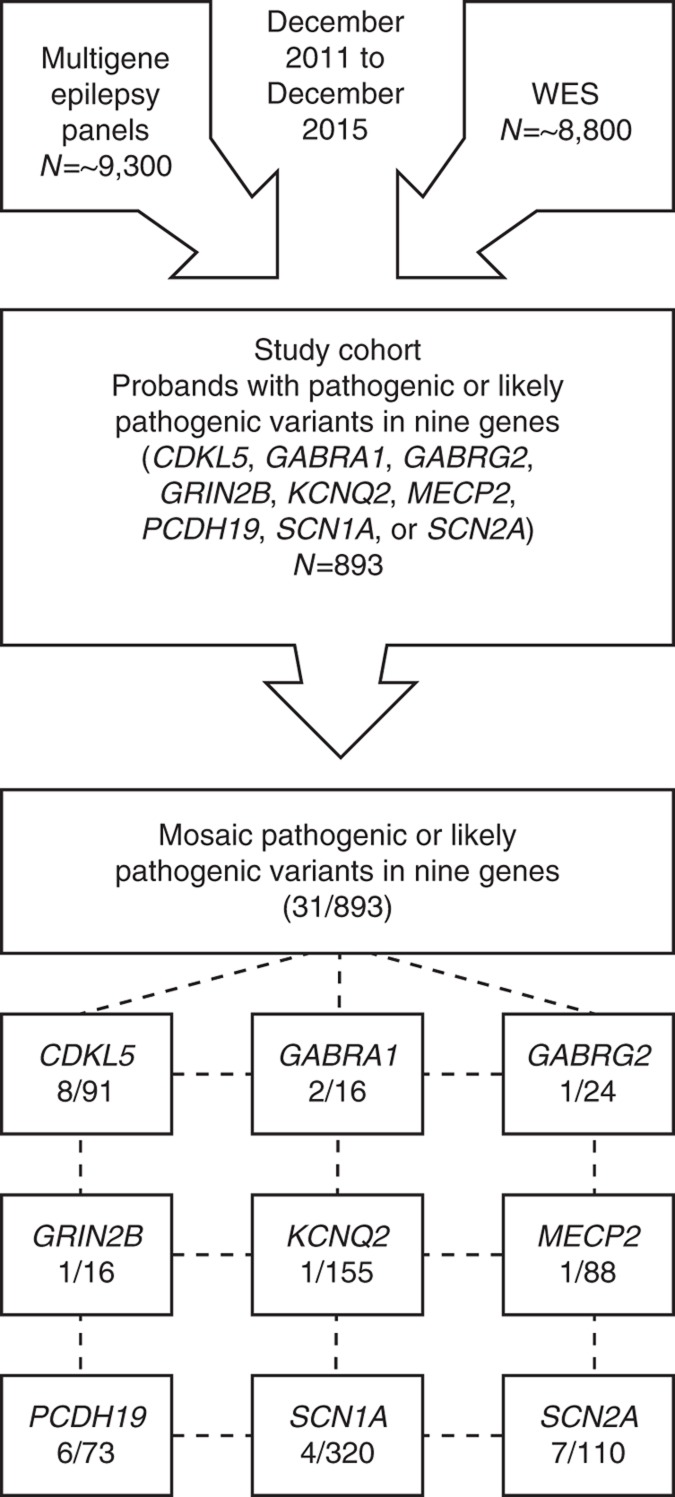
**Flow chart of testing.** Schematic overview of the breakdown of testing for the study cohort. WES, whole-exome sequencing.

The cohort included 893 probands, referred from December 2011 to December 2015, who had undergone NGS testing for either a panel of up to 70 epilepsy-related genes or whole-exome sequencing (WES) and were found to be positive for a pathogenic or likely pathogenic variant in one of the following genes: *CDKL5*, *GABRA1*, *GABRG2*, *GRIN2B*, *KCNQ2*, *MECP2*, *PCDH19*, *SCN1A*, or *SCN2A* (Figure 1). These nine genes were selected because mosaic pathogenic and likely pathogenic variants were observed in one or more probands with epilepsy by NGS and frequencies of mosaicism for these genes have not been previously reported, to our knowledge. In addition to epilepsy, clinical indications for testing often included other neurodevelopmental features such as developmental delay, intellectual disability, learning disability, developmental regression, autism spectrum disorder, behavioral/psychiatric abnormalities, hypotonia, microcephaly/macrocephaly, and movement disorders. Clinical information, family history, and ethnicity were provided using a clinical checklist and/or clinic notes submitted by the referring clinicians, who were primarily neurologists or geneticists working in private practice, private hospitals, or university-based medical centers. The majority of patients resided in the United States or Canada. In a subset of probands with heterozygous or hemizygous pathogenic or likely pathogenic variants, parental mosaicism was evaluated either by NGS or targeted Sanger sequencing. Informed consent for clinical testing was obtained by the referring clinicians.

### Next-generation sequencing

A targeted capture panel (ranging from 18 to 70 genes) or WES was used to investigate genes associated with epilepsy-related disorders. For all panels, targeted regions were enriched from genomic DNA extracted from blood using either multiplex polymerase chain reaction (PCR) by Raindance Technologies (Raindance Technologies, Lexington, MA) or RNA base hybridization capture using a custom designed library and SureSelect kit (Agilent Technologies, Santa Clara, CA) following the manufacturer’s instructions. Specifically, for libraries generated with Raindance Technologies (Billerica, MA), DNA was sheared on a Covaris instrument (Woburn, MA) to produce 5-kb fragments that were purified using the Qiagen QIAQuick (Hilden, Germany) mini-elute kit. DNA fragments were merged with PCR primers in microdroplets and transferred to PCR tubes for PCR amplification. The amplified products were enzymatically fragmented and tagged with adaptors using the standard Nextera protocol (Illumina, San Diego, CA). A final PCR enrichment amplification was performed at 65°C for 15 cycles. Products were purified using the Qiagen QIAQuick mini-elute kit. For Agilent capture processing, DNA was sheared into approximately 400-bp fragments, which were then repaired, ligated to adaptors, and purified for subsequent PCR amplification. Amplified products were then captured by biotinylated RNA library baits in solution following the manufacturer’s instructions. Bound DNA was isolated with streptavidin-coated beads and reamplified.

The final library products for both methods were sequenced using the Illumina HiSeq 2000 or 2500 sequencing system with either 50-bp single-end or 100-bp paired-end reads, respectively (Illumina, San Diego, CA), following manufacturer’s instructions and described previously.^15^ All targeted gene regions were created as custom contigs based on RefSeq gene sequences. For Raindance, reads were mapped to only the custom contig targets, while for capture, to avoid misalignment of any genomic carryover, read alignment used the curated gene regions plus a masked version of hg19 to filter any genomic carryover. Local realignment around insertion/deletion sites was performed using the Genome Analysis Toolkit v1.6.^16^ Regions of interest were defined as the exon and flanking noncoding sequences. All samples were sequenced to an average coverage of at least 1200 ×. Positions within the region of interest covered at less than 15 × were compensated for by conventional Sanger DNA sequencing using an ABI 3730 (Life Technologies, Carlsbad, CA) and standard protocols. All variants called within the region of interest by any of these programs were analyzed, and all novel variants that appeared real based on manual inspection of NGS alignments and all previously classified, nonbenign variants were confirmed by conventional Sanger DNA sequence analysis with a new DNA preparation.

For WES, genomic DNA was extracted from whole blood of affected probands and, when available, their parents and other similarly affected relatives. Next-generation sequencing was performed on exon targets captured using the Agilent SureSelect Human All Exon V4 (SS V4) or Clinical Research Exome kit (Agilent Technologies) to an average target depth of more than 100 ×. The sequencing methodology, data processing pipeline, and variant filtering and interpretation protocol have been described previously.^17^ All clinically relevant variants were confirmed by conventional Sanger DNA sequence analysis with a new DNA preparation.

The read depth varied based on the specific NGS method, Raindance, custom capture, or whole exome sequencing (Supplementary Table S1 online and Supplementary Figure S1). Supplementary Figure S1 shows that the per-target coverage is more consistent for capture-based methods. The capture panel provides the best coverage for consistent mosaic variant calling with nearly all targets consistently covered to >100 × and most targets consistently covered to >200 ×. While the Raindance panel provides higher coverage for some targets, its coverage uniformity is poor.

### Variant analysis and interpretation

Mosaicism was hypothesized when variants in autosomal genes and X-linked genes in females were observed at a lower-than-expected ratio of variant to wild-type NGS reads and exhibited unequal amplification by Sanger sequencing. For males, mosaicism was suspected for X-linked genes when both variant and wild-type alleles were observed and when there was no indication of a sex chromosome aneuploidy based on either concurrent exon-level array comparative genomic hybridization data or prior chromosome analysis by karyotype.

The general assertion criteria for variant classification are publicly available on the GeneDx ClinVar submission page (http://www.ncbi.nlm.nih.gov/clinvar/submitters/26957/). Variants were categorized using the five-tier classification system recommended by the American College of Medical Genetics and Genomics. Clinical variant interpretation was conducted in the context of the proband’s phenotype when available. Likely pathogenic variants have a high probability of being pathogenic. Pathogenic and likely pathogenic variants were used for the analysis of this manuscript. For the sake of simplicity, all of the qualifying variants are referred to as “pathogenic” in this manuscript, although many are classified in ClinVar as “likely pathogenic.”

## Results

### Mosaicism in probands

Molecular diagnostic testing for epilepsy-related disorders utilizing NGS yielded positive diagnostic results in 893 probands, including pathogenic variants in nine genes analyzed (*CDKL5*, *GABRA1*, *GABRG2*, *GRIN2B*, *KCNQ2*, *MECP2*, *PCDH19*, *SCN1A*, and *SCN2A*) (Figure 1). For the multigene NGS panels, more than 9,300 probands were tested in the specified time period, and these nine genes accounted for approximately 57% of the overall positive cases. Among all positive cases identified through the multigene NGS panels, missense variants accounted for the majority (52%) of pathogenic variants detected; frameshift accounted for 23%, nonsense 14%, splice 10%, and insertion/deletion 1%. Among probands with positive results in these nine genes (*n* = 893, Supplementary Figure S1), 31 demonstrated a lower-than-expected ratio of variant to wild-type NGS reads, which is indicative of mosaicism in the nine selected genes. The mean read depth at the site of pathogenic changes was 1,299 (range: 42–3,444 reads) (Table 1). The level of observed mosaicism ranged from 9 to 40% of sequencing reads in probands with autosomal dominant disorders and females with X-linked disorders. Males with an X-linked disorder had mosaic variants in 8–87% of sequencing reads (Table 1). In this study, the breakdown of the types of mosaic variants in these nine genes were missense (68%), nonsense (16%), frameshift (10%), and splice site (6%) variants (Table 1). The mean read depth across the nine genes with mosaic pathogenic variants is provided in Supplementary Table S1 for each of the different testing methods. Density plots of per-sample, per-target coverage for each of the four methods are illustrated in Supplementary Figure S1.

The highest frequency of mosaicism among positive probands was observed for two X-linked genes, 8.8% for *CDKL5* (0.088; 95% CI, 0.039–0.166) and 8.2% for *PCDH19* (0.082; 95% CI, 0.031–0.170) (Table 2). Of the individuals with pathogenic variants in *CDKL5*, 21% (19/91) were male. Additionally, 75% (6/8) of mosaic pathogenic variants identified in *CDKL5* were in affected males noted to have epilepsy and/or developmental delay, and 25% (2/8) were in affected females with epilepsy and developmental delay. Although *PCDH19*-related epilepsy is an X-linked dominant disorder almost exclusively affecting females, five males were found to be mosaic for a pathogenic variant in the *PCDH19* gene and presented with epilepsy and/or developmental delay, similar to females with *PCDH19* pathogenic variants.

Mosaic pathogenic variants were observed in 6.4% of patients with pathogenic variants in *SCN2A* (0.064; 95% CI, 0.026–0.128) (Table 2). All patients with mosaic *SCN2A* pathogenic variants had epilepsy and many (5/7) were also noted to have developmental delay based on limited clinical information provided. Additionally, an unusual finding of double mosaicism, previously referred to as triple mosaicism,^18^ was identified in one patient who was mosaic for two different pathogenic variants at the same nucleotide position in the *SCN2A* gene, with each sequence change having different variant allele frequencies (Table 1). The frequency of mosaicism in *SCN1A* (0.013; 95% CI, 0.003–0.032) was significantly lower than in *SCN2A* (*P* < 0.01; two-tailed Fisher’s exact test) (Table 2).

Of note, we also detected mosaic pathogenic variants in several additional epilepsy-related genes including some of the GABA receptor genes, *GABRA1* (0.125; 95% CI, 0.016–0.384) and *GABRG2* (0.042; 95% CI, 0.001–0.211); *GRIN2B* (0.063; 95% CI, 0.002–0.302), which encodes the glutamate-binding NR2B subunit of the *N*-methyl-d-aspartate receptor; the potassium channel gene *KCNQ2* (0.006; 95% CI, 0.001–0.035); and *MECP2* (0.011; 95% CI, 0.0003–0.062) in a male with atypical Rett syndrome (Table 2).

These data indicate that the overall frequency of mosaic pathogenic variants in the two genes, *CDKL5* and *PCDH19* (0.085; 95% CI, 0.048–0.139), was significantly higher than the overall frequency of mosaic pathogenic variants in the other seven genes combined, *GABRA1*, *GABRG2*, *GRIN2B*, *KCNQ2*, *MECP2*, *SCN1A*, and *SCN2A* (0.023; 95% CI, 0.014–0.037), (*P* < 0.01; two-tailed Fisher’s exact test).

### Mosaicism in parents

Parental testing was performed for probands undergoing trio-based WES and recommended for all probands with positive results identified via panel testing. For probands tested by multigene NGS panels and identified to have pathogenic variants in *CDKL5*, *GABRA1*, *GABRG2*, *GRIN2B*, *KCNQ2*, *MECP2*, *PCDH19*, *SCN1A*, or *SCN2A*, 290 sets of parents underwent targeted testing for the variant by Sanger sequencing. Of these, 237 (81.7%) were determined to be de novo and 53 (18.3%) were inherited (Table 2). Similarly, for probands tested by WES trio with a pathogenic variant in one of these nine genes, the overall frequency of de novo variants was 84.8% (89/105) (Table 2). For parents tested either by targeted Sanger sequencing after the proband had a positive result by multigene NGS panel or by WES trio testing, we observed that 12 parents were suspected to have mosaic variants in either *SCN1A* (6), *SCN2A* (3), *KCNQ2* (2), or *MECP2* (1) (Table 3). Clinical information was not provided for one parent, but it was provided for the other 11 parents. Based on the clinical information provided, 27% (3/11) of these parents were clinically affected with clinical features consistent with the disorders associated with these genes (Table 3). However, two of the three affected parents were reported to have milder or later-onset symptoms compared with their children. The remaining 73% (8/11) of the parents who carried a mosaic pathogenic variant and for whom clinical information was provided were reportedly unaffected (Table 3). In addition, we observed one case of suspected germ-line mosaicism for an *SCN1A* pathogenic variant in a family with two affected siblings (Table 3). This *SCN1A* variant was undetectable via targeted Sanger sequencing of DNA obtained from blood from both parents.

## Discussion

Mosaicism has been reported in the literature in genes associated with epilepsy and neurodevelopmental disorders. However, for most genes, information has been limited to case reports. While these publications provide evidence that mosaicism can occur in genes causing epilepsy, the frequency of mosaic findings is not known for most genes. Based on our analyses, mosaic pathogenic variants were identified at an overall frequency of 3.5% (0.035; 95% CI, 0.024–0.049) in nine genes associated with epilepsy-related disorders (Table 2). Additionally, we report on the observed occurrence of mosaic pathogenic variants in several epilepsy-related genes. The *CDKL5* gene encodes a serine/threonine protein kinase that is highly expressed in the brain, mediates phosphorylation of *MECP2*, and is associated with variable clinical phenotypes.^19, 20, 21^ *CDKL5*-related disorders are X-linked dominant and often result in male lethality or, less commonly, in a severe phenotype with early-onset epileptic encephalopathy and intellectual disability. Mosaic pathogenic variants have been reported occasionally in affected females and males, but may be more prevalent than previously understood.^4, 9, 10, 20, 22, 23^ In our cohort, mosaic variants in *CDKL5* account for nearly 9% of all *CDKL5*-positive cases. Additionally, of the males who were positive for a pathogenic variant in *CDKL5*, 32% were found to be mosaic.

The *PCDH19* gene encodes protocadherin-19, a calcium-dependent cell–cell adhesion molecule primarily expressed in the brain. Pathogenic variants in *PCDH19* cause a type of early infantile epileptic encephalopathy that is sometimes referred to as epilepsy and mental retardation limited to females. This condition follows an unusual X-linked inheritance pattern in which females are affected while males who are hemizygous for a pathogenic variant are unaffected carriers. Although the mechanism of disease is not completely understood, cellular interference has been speculated.^7, 24^ Mosaic pathogenic variants have been reported in affected males and females.^7, 8, 11, 24, 25, 26^ We identified mosaic pathogenic variants in *PCDH19* in over 8% of patients with a *PCDH19*-related disorder. As expected based on the hypothesized dominant-negative etiology for *PCDH19*-related disorders, all males with a mosaic pathogenic *PCDH19* variant were found to be clinically affected. Additionally, two affected males in our cohort were hemizygous for pathogenic variants in the *PCDH19* gene (one frameshift and one missense); these patients had no evidence of wild-type allele in the DNA sample extracted from blood. It is possible that the phenotype in these male patients was not caused by the pathogenic *PCDH19* variants identified and the patients were coincidentally found to be carriers of the variants. Another possibility is that these male patients are mosaic, but we were unable to detect the wild-type allele in the provided samples extracted from blood. A prior publication included a report of a male who appeared hemizygous for a pathogenic *PCDH19* variant with no evidence of mosaicism in one tissue (blood), while a sample from another tissue (skin) revealed the presence of mosaicism.^7^ In the patients reported here, alternate tissue types were not submitted, limiting certain conclusions.

The *SCN2A* gene encodes the alpha subunit of a neuronal sodium channel, which mediates normal neuronal firing.^27^ Pathogenic variants in *SCN2A* cause benign familial neonatal–infantile seizures, neonatal epileptic encephalopathy, and other types of epilepsy.^27, 28^ Mosaic pathogenic variants in *SCN2A* have only been reported in the literature in two cases to date.^29, 30^ Paternal germ-line mosaicism for an *SCN2A* pathogenic variant that was transmitted to two affected half-siblings has also been reported.^31^ Our data demonstrate that more than 6% of patients with a pathogenic *SCN2A* variant were mosaic.

The *SCN1A* gene encodes the alpha subunit of a neuronal voltage-gated sodium channel that regulates the excitability of neurons.^32^ Pathogenic variants in *SCN1A* cause a variety of epilepsy phenotypes, ranging from simple febrile seizures to severe infantile epileptic encephalopathies. The frequency of mosaic pathogenic variants detected in *SCN1A* in probands was 1.3% the overall frequency of mosaicism in *SCN1A* that we observed for families with pathogenic variants in the *SCN1A* gene was 3.1% (10/320). In addition to the four probands found to harbor mosaic pathogenic variants in *SCN1A*, there were six instances of parental mosaicism for *SCN1A* pathogenic variants. The majority of parents with mosaic *SCN1A* pathogenic variants were reportedly unaffected. These data support previous reports that parental mosaicism in *SCN1A* may be more common than previously thought.^33^ In addition, other investigators have indicated that parental mosaicism was not detected in their testing cohort by targeted Sanger sequencing, and that more sensitive tools for detecting low-level mosaicism in parents could allow for more accurate diagnosis.^33, 34, 35^

Mosaic pathogenic variants were also observed in the *GABRA1*, *GRIN2B*, *GABRG2*, *MECP2*, and *KCNQ2* genes. Although previous case studies have included reports of mosaic pathogenic variants in *KCNQ2* and *MECP2* in probands and/or their parents, ours is, to our knowledge, the first report of mosaic variants in the *GABRA1*, *GABRG2*, and *GRIN2B* genes. Although these genes had a relatively high frequency of mosaicism, limited data were available due to the small number of positive cases with pathogenic variants in these genes (Table 2).

We report parental mosaicism for pathogenic variants in the *SCN1A*, *SCN2A*, *KCNQ2*, and *MECP2* genes. Parental mosaicism in these genes has been reported in unaffected, mildly affected, and more severely affected parents; therefore, the likelihood of parental mosaicism appears to be difficult to predict based on parental phenotype.^5, 34, 36, 37, 38, 39^ In our data set, of the parents who were identified as mosaic carriers of a pathogenic variant, and for whom we received clinical information, 73% (8/11) were reported to have no related neurological features. The parents identified as mosaic carriers of a pathogenic variant who were also reported to have a neurodevelopmental phenotype typically had features that were less severe than the features reported for their affected child. Taken together, our NGS-based data suggest that parental mosaicism may be a relatively frequent and underappreciated occurrence for pathogenic variants in genes causing epilepsy, and that the possibility of parental mosaicism should be considered when providing counseling about reproductive risks to parents of a child with a pathogenic variant in an epilepsy-related gene. Targeted parental testing is routinely performed in clinical laboratories utilizing Sanger sequencing, which is a qualitative assay and therefore has limited sensitivity in detecting mosaicism. NGS with high read depth should be considered for parental samples when testing for variants in genes with a high frequency of mosaic pathogenic variants for better sensitivity of low-level mosaicism.

In summary, individuals with epilepsy who previously tested negative for pathogenic variants in these genes by Sanger sequencing may benefit from a repeat analysis using NGS, which is more sensitive in the detection of mosaic variants. For any proband with a pathogenic variant in these genes, targeted testing of both parents is indicated and should be performed by NGS or another quantitative assay to better evaluate for possible parental mosaicism and more accurately estimate the recurrence risk. A careful and systematic review of laboratory diagnostic data may reveal that the frequency of mosaicism in genes associated with other disorders is higher than currently reported. To determine this, the methodology and threshold of variant calls would need to be optimized for consistent detection of low-level mosaicism and multiple tissue types would need to be screened. This endeavor could possibly be accomplished by multicenter collaborations and/or clinic–research collaborations.

## Figures and Tables

**Table 1:** Cases with mosaic pathogenic variant(s)

**Gene**	**Gender**	**Variant (hg19 position; coding DNA; protein)**	**Type of variant**	**Testing method**	**% of variant reads**	**Coverage (read depth at variant position)**
*Autosomal dominant inheritance*						
* GABRA1*	Male	chr5:161317988 T>C; c.788 T>C; p.Met263Thr	Missense	Raindance	11.7%	803
* GABRA1*	Male	chr5:161317999 C>A; c.799 C>A; p.Leu267Ile	Missense	Raindance	18.6%	483
* GABRG2*	Male	chr5:161522522 C>A; c.281 C>A; p.Thr94Lys	Missense	Raindance	14.4%	667
* GRIN2B*	Male	chr12:13720105 A>C; c.2452 A>C; p.Met818Leu	Missense	Custom capture	24.4%	2,574
* KCNQ2*	Female	chr20:62044866 T>A; c.1700 T>A; p.Val567Asp	Missense	Custom capture	30.2%	463
* SCN1A*	Male	chr2:166852531 C>T; c.4573 C>T; p.Arg1525Ter	Nonsense	Raindance	9.2%	1,021
* SCN1A*	Male	chr2:166915131 T>A; c.332 T>A; p.Leu111Ter	Nonsense	Custom capture	20.2%	3,406
* SCN1A*	Female	chr2:166903453 T>C; c.1204 T>C; p.Phe402Leu	Missense	Custom capture	24.7%	3,107
* SCN1A*	Female	chr2:166929992 A>-; c.140delA; p.Asn47MetfsTer45	Frameshift	Raindance	26.0%	412
* SCN2A*	Male	chr2:166210777 G>A; c.2995 G>A; p.Glu999Lys	Missense	Raindance	11.6%	627
* SCN2A*	Male	chr2:166201203 C>G; c.2701 C>G; p.Gln901Glu	Missense	Custom capture	13.0%	3,444
* SCN2A*	Male	chr2:166198881 G>A; c.2464 G>A; p.Gly822Ser	Missense	WES	14.7%	95
* SCN2A*	Female	chr2:166237619 T>A; c.4463 T>A; p.Ile1488Asn	Missense	Raindance	16.8%	1,413
* SCN2A*	Female	chr2:166201153 T>A; c.2651 T>A; p.Leu884His	Missense	Raindance	20.3%	488
* SCN2A*	Female	chr2:166165888 G>A; c.632 G>A; p.Gly211Asp	Missense	Custom capture	22.1%	961
* SCN2A*^a^	Male	chr2:166231223 T>C, T>A c.4001 T>C, c.4001 T>A; p.Ile1334Thr, p.Ile1334Asn	Two missense at same nucleotide position	Custom capture	27.1% 39.5%	3,320 3,320
						
*X-linked inheritance*						
* CDKL5*	Female	chrX:18613489 C>T; c.766 C>T; p.Gln256Ter	Nonsense	Custom capture	10.1%	2,353
* CDKL5*	Male	chrX:18602452 G>A; c.533 G>A; p.Arg178Gln	Missense	WES	14.3%	42
* CDKL5*	Female	chrX:18600056 A>G; c.449 A>G; p.Lys150Arg	Missense	Raindance	16.0%	562
* CDKL5*	Male	chrX:18627690 G>A; c.2152 G>A; p.Val718Met	Missense	Raindance	25.3%	2,961
* CDKL5*	Male	chrX:18598085 C>T; c.400 C>T; p.Arg134Ter	Nonsense	Raindance	31.1%	380
* CDKL5*	Male	chrX:18622719 C>T; c.1675 C>T; p.Arg559Ter	Nonsense	WES	36.1%	180
* CDKL5*	Male	chrX:18528948 C>A; c.73 G>A; p.Gly25Arg	Missense	Raindance	41.2%	284
* CDKL5*	Male	chrX:18627686-18627687 CA>-; c.2148_2149delCA; p.Tyr716Ter	Frameshift	Raindance	56.3%	895
* MECP2*	Male	chrX:153297671 G>A; c.364 G>A; p.Val122Met	Missense	Custom capture	31.4%	1,797
* PCDH19*	Male	chrX:99661637-99661640 CTCT>-; c.1956_1959delCTCT; p.Ser653ProfsTer6	Frameshift	Custom capture	8.1%	1,551
* PCDH19*	Female	chrX:99662962 G>C; c.634 G>C; p.Asp212His	Missense	Raindance	12.5%	2,556
* PCDH19*	Male	chrX:99605642 T>C; c.2534+2 T>C	Splice	Raindance	22.2%	189
* PCDH19*	Male	chrX:99661447 T>C; c.2147+2 T>C	Splice	Custom capture	43.6%	1,338
* PCDH19*	Male	chrX:99661723 A>G; c.1873 A>G; p.Arg625Gly	Missense	Raindance	83.1%	688
* PCDH19*	Male	chrX:99663226 G>C; c.370 G>C; p.Asp124His	Missense	Raindance	87.2%	1,219

WES, whole-exome sequencing.

a Single patient is mosaic for two different nucleotide substitutions at same codon, known as double mosaicism.

**Table 2:** Frequency of mosaicism for each reported gene considering probands who were mosaic for a pathogenic or likely pathogenic variant in the corresponding gene

**Gene**	**Disease/phenotype**	**Primary transcript**	**Percent of probands with mosaic variants**	**No. of mosaic variants/total no. of pathogenic and likely pathogenic variants**	**95% CI**	**Percent of de novo variants detected by multigene panels when both parents tested by targeted dideoxy sequencing (no. of de novo variants/no. of cases tested)**	**Percent of de novo variants for positive cases when tested via trio-based WES**
*CDKL5*	Atypical RS, EIEE	NM_003159.2	8.8%	8/91	0.039–0.166	100% (26/26)	100% (6/6)
*PCDH19*	EIEE (EFMR)	NM_001184880.1	8.2%	6/73	0.031–0.170	75.9% (22/29)	40% (2/5)
*SCN2A*	EIEE, BFNIS, GEFS+, IS, ICE	NM_021007.2	6.4%	7/110	0.026–0.128	89.8% (44/49)	95.2% (20/21)
*SCN1A*	GEFS+, ICE-GTCS, EIEE, SMEI, DS	NM_001165963.1	1.3%	4/320	0.003–0.032	75.5% (74/98)	73.7% (14/19)
*GABRA1*	OS, DS, IS, GEFS+, JME, CAE	NM_000806.5	12.5%	2/16	0.016–0.384	100% (8/8)	100% (2/2)
*GRIN2B*	EIEE, ID	NM_000834.3	6.3%	1/16	0.002–0.302	50% (1/2)	100% (12/12)
*GABRG2*	FS, FS with CAE, GEFS+	NM_000816.3	4.2%	1/24	0.001–0.211	73.3% (11/15)	50% (1/2)
*MECP2*	RS, atypical RS	NM_004992.3	1.1%	1/88	0.0003–0.062	77.8% (7/9)	81% (17/21)
*KCNQ2*	BFNS, EIEE	NM_172107.2	0.6%	1/155	0.001–0.035	81.5% (44/54)	88.2% (15/17)
Total			3.5%	31/893	0.024–0.049	81.7% (237/290)	84.8% (89/105)

BFNIS, benign familial neonatal–infantile seizures; BFNS, benign familial neonatal seizures; CAE, childhood absence epilepsy; DS, Dravet syndrome; EFMR, epilepsy and mental retardation limited to females; EIEE, early infantile epileptic encephalopathy; FS, febrile seizures; GEFS+, genetic (generalized) epilepsy with febrile seizures plus; ICE, intractable childhood epilepsy; ICE-GTCS, intractable childhood epilepsy with generalized tonic–clonic seizures; ID, intellectual disability; IS, infantile spasms; JME, juvenile myoclonic epilepsy; OS, Ohtahara syndrome; RS, Rett syndrome; SMEI, severe myoclonic epilepsy of infancy; WES, whole-exome sequencing.

95% confidence interval (CI) is provided.

**Table 3:** Clinical information for cases of parental mosaicism

**Gene**	**Variant (hg19 position; coding DNA; protein)**	**Type of variant**	**Percent of variant reads**	**Coverage (read depth at variant position)**	**Parent: clinical information**	**Parent: onset of seizures**	**Proband: onset of seizures**
*SCN1A*	chr2:166929897 G>A; c.235 G>A; p.Asp79Asn	Missense	12.6%	495	Unknown	Unknown	Unknown
*KCNQ2*	chr20:62076020 C>T; c.682 C>T; p.His228Tyr	Missense	27.1%	266	Unaffected	N/A	Neonatal
*MECP2*	chrX:153296354 C>T; c.925 C>T; p.Arg309Trp	Missense	19.9%	540	Unaffected	N/A	Infancy
*SCN1A*	chr2: 166909392 C>T; c.664 C>T; p.Arg222Ter	Nonsense	6.0%	847	Unaffected	N/A	Infancy
*SCN1A*	chr2:166852557 C>A; c.4547 C>A; p.Ser1516Ter	Nonsense	14.2%	690	Unaffected	N/A	Unknown
*SCN1A*	chr2:166929950 T>C; c.182 T>C; p.Leu61Pro	Missense	20.2%	1,011	Unaffected	N/A	Infancy
*SCN1A*	chr2:166850727 C>A; c.4781 C>A; p.Ser1594Tyr	Missense	15.8%	38	Unaffected	N/A	Early childhood
*SCN1A*^a^	chr2:166894519 G>A; c.2713 G>A; p.Ala905Thr	Missense	N/A	N/A	Unaffected	N/A	Unknown
*SCN2A*	chr2: 166237633-166237635 GAA>-c.4477_4479delGAA; p.Glu1493del	In-frame deletion	9.0%	885	Unaffected	N/A	Infancy
*SCN2A*	chr2:166245211 G>A; c.4895 G>A; p.Arg1632Lys	Missense	16.4%	764	Unaffected	N/A	Neonatal
*KCNQ2*	chr20:62073785 T>A; c.790 T>A; p.Tyr264Asn	Missense	14.0%	172	Affected (mild compared with proband)	Infancy	Neonatal
*SCN1A*	chr2:166848302 T>C; c.5483 T>C; p.Leu1828Ser	Missense	18.1%	533	Affected (more severe compared with proband)	Neonatal	Unknown (asymptomatic in infancy)
*SCN2A*	chr2:166245224 C>G; c.4908 C>G; p.Ile1636Met	Missense	7.6%	885	Affected (mild compared with proband)	Infancy	Neonatal

N/A, not applicable.

a Suspected germ-line mosaicism: both parents were negative by targeted dideoxy sequencing and two affected offspring were heterozygous for variant.
