# Supplementary material for: High frequency of mosaic pathogenic variants in genes causing epilepsy-related neurodevelopmental disorders
Source: Genet Med. 2017 Aug 24;20(4):403–10. doi: 10.1038/gim.2017.114 (PMC5895461; doi:10.1038/gim.2017.114)
Supplement: Supplementary Table S1 [file gim2017114x2.pdf]

Table S1. Mean coverage (read depth) of genes by various NGS methods

	Mean coverage (read depth) of NGS methods			
**Gene**	**Panel: Raindance (multiplex PCR)**	**Panel: Capture**	**WES V1 (SS V4)**	**WES V2 (CRE)**
CDKL5	640	1,070	131	166
PCDH19	1,020	930	176	153
SCN2A	1,140	1,170	102	215
SCN1A	1,280	1,200	99	208
GABRA1	670	1,430	109	190
GRIN2B	N/A	1,350	160	163
GABRG2	630	1,460	114	248
MECP2	2,020	880	182	154
KCNQ2	1,090	1,150	91	81

CRE, Clinical Research Exome; SS V4, SureSelect Human All Exon V4.
